# *Wolbachia*: A bacterial weapon against dengue fever- a narrative review of risk factors for dengue fever outbreaks

**DOI:** 10.1016/j.nmni.2025.101578

**Published:** 2025-03-08

**Authors:** Sahel Safaei, Mozhgan Derakhshan-sefidi, Amirmohammad Karimi

**Affiliations:** a Department of Bacteriology, Faculty of Medical Sciences, Tarbiat Modares University, Tehran, Iran; b Lorestan University of Medical Sciences, Khorramabad, Iran

**Keywords:** *Wolbachia*, Dengue fever, Public health, Tropical infections, New emerging infection, Epidemiological risk factors

## Abstract

Arboviruses constitute the largest known group of viruses and are responsible for various infections that impose significant socioeconomic burdens worldwide, particularly due to their link with insect-borne diseases. The increasing incidence of dengue fever in non-endemic regions underscores the urgent need for innovative strategies to combat this public health threat. *Wolbachia*, a bacterium, presents a promising biological control method against mosquito vectors, offering a novel approach to managing dengue fever. We systematically investigated biomedical databases (PubMed, Web of Science, Google Scholar, Science Direct, and Embase) using “AND” as a Boolean operator with keywords such as “dengue fever,” “dengue virus,” “risk factors,” “*Wolbachia*,” and “outbreak.” We prioritized articles that offered significant insights into the risk factors contributing to the outbreak of dengue fever and provided an overview of *Wolbachia*'s characteristics and functions in disease management, considering studies published until December 25, 2024.

Field experiments have shown that introducing *Wolbachia*-infected mosquitoes can effectively reduce mosquito populations and lower dengue transmission rates, signifying its potential as a practical approach for controlling this disease.

## 1 Background

**Fig. 1:**
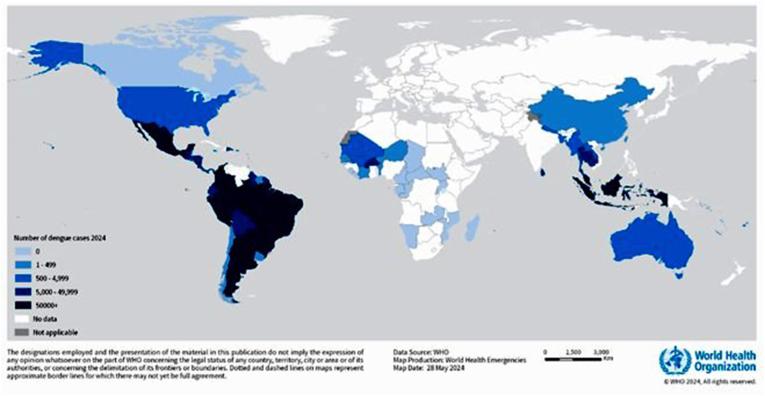
The geographical spread of dengue cases reported to the World Health Organization (WHO) from January to April 2024 (https://www.who.int/emergencies/disease-outbreak-news/item/2024-DON518).

*Wolbachia (W.)*, a naturally occurring bacterial symbiont found in approximately half of all insect species, presents a viable and innovative strategy for managing mosquito vectors of arboviruses like dengue fever virus in non-endemic areas [1]. *Wolbachia* is an α-proteobacterium belonging to the family Rickettsiaceae [2]. It was initially discovered in the reproductive cells of the mosquito *Culex pipiens* and possesses the capability for both vertical and horizontal transmission (1924, *Wolbachia pipientis*) [[3], [4], [5]]. *Wolbachia's* supergroups exert significant influences over arthropods and filarial nematodes including parthenogenesis, feminization of genetic males, male embryo death, and cytoplasmic incompatibility (CI) [6]. Additionally, *Wolbachia* influences reproduction by causing non-viable offspring when an uninfected female mosquito mates with a male carrying this bacterial infection [7]. *Wolbachia*'s metabolic dependencies underscore the complex interactions between this bacterium and its hosts. Notably, these bacteria possess incomplete metabolic pathways for synthesizing essential amino acids. As a result, they typically rely on parasitizing to acquire these vital nutrients [4,8,9]. As a mosquito-borne illness, dengue faces considerable challenges in control efforts that rely on insecticides. High costs and the development of resistance among mosquito populations significantly undermine the effectiveness of these programs, complicating efforts to manage mosquito numbers and mitigate transmission. Scientists are exploring the potential of *Wolbachia*-based strategies to combat various mosquito-borne illnesses. This innovative approach harnesses the bacterium *Wolbachia* to render mosquitoes immune to certain diseases, including dengue fever virus [10]. Studies revealed mosquitoes infected with *Wolbachia* are resistant to dengue fever, and this feature can be maternally transmitted. The ability to pass this trait through generations provides a promising long-term solution [11]. Recent data indicates a staggering rise in dengue cases, with over 12.7 million reported between January and September 2024—almost double the 6.5 million cases documented in 2023—and approximately 8791 deaths attributed to the illness (https://www.weforum.org/).This alarming trend underscores the urgent need for enhanced public health strategies to combat the spread of dengue fever effectively (https://www.cdc.gov/dengue) (Fig. 1). Numerous field investigations have shown the practicality and efficacy of incorporating *Wolbachia* into indigenous mosquito populations [12,13]. Artificial intelligence and mathematical models have been extensively studied to predict the spread of *Wolbachia* among mosquito populations. Given the numerous variables affecting dengue fever transmission worldwide, researchers are investigating novel approaches using bacteria to control mosquitoes that transmit this disease and other vector-borne illnesses [[14], [15], [16], [17], [18]]. Mosquitoes, such as *Aedes aegypti* (*Stegomyia aegypti*) and *Aedes albopictus* (*Stegomyia albopictus*) typically have a lifespan of 10–22 days, influenced by environmental factors like temperature and humidity. These mosquito species are known vectors for various diseases, including dengue fever [19,20]. Individuals of both sexes, male and female mosquitoes, that infected with *Wolbachia* can successfully mate with non-infected partners of the opposite sex or a compatible strain of *Wolbachia*. However, the reproductive outcome of such pairings is contingent upon the specific *Wolbachia* strain harbored by the male [21]. CI is a complex phenomenon that primarily impacts male offspring. CI occurs when a male carrying a particular *Wolbachia* strain mates with a female lacking the bacterium or possessing an incompatible strain, embryonic development may be compromised or halted. Conversely, if the female carries a compatible *Wolbachia* strain, normal embryonic development proceeds [[22], [23], [24], [25], [26]]. Certain strains of *Wolbachia* have been shown to shorten mosquitoes' lifespan. It is significant to note that *Wolbachia* seems to diminish the virulence of dengue infections within these mosquitoes [27,28]. The deployment of *Wolbachia*-infected mosquitoes has proven to be a viable strategy for disease management, as evidenced by field trials that demonstrate significant reductions in mosquito populations and dengue transmission rates [29].

## 2 Methodology

To gain a comprehensive understanding of dengue fever and the role of *Wolbachia* as a novel control agent, we conducted an extensive literature search using databases such as PubMed, Web of Science, Google Scholar, Science Direct, and Embase. A structured approach was employed, utilizing “AND” or “OR” as Boolean operators with several keywords including “dengue fever,” “dengue virus,” “risk factors,” “*Wolbachia*,” and “outbreak.” We focused on articles that provided significant insights into the clinical presentation, transmission, and epidemiology of the dengue virus and the effects of *Wolbachia* in controlling dengue fever, considering studies published until December 25, 2024. Both seminal studies and recent case reports were included to reflect the evolution of knowledge and recent advancements in the field. References from the selected articles were cross-checked to ensure data authenticity. Exclusion criteria comprised articles irrelevant to our research objectives, those not written in English, and poorly controlled trials.

## 3 The risk factors contributing to the outbreak of dengue fever

**Fig. 2:**
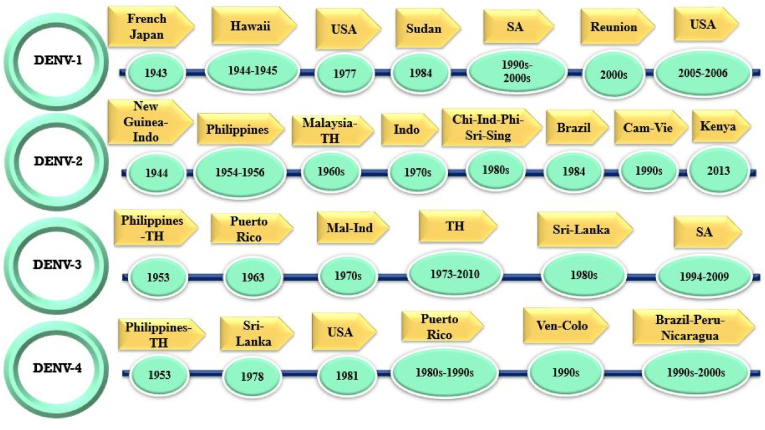
Distribution of occurrences for each dengue virus (DENV) type [33]. SA: Saudi Arabia, TH: Thailand, Chi: China, Ind: India, phi: Philippines, Sri: Sri Lanka, Sing: Singapore, Cam: Cambodia, Vie: Vietnam, Mal: Malaysia, Indo: Indonesia, Ven: Venezuela, Colo: Colombia.

A global shift in the pattern of dengue fever outbreaks as an arboviral disease has been noted recently. This flavivirus infection is primarily transmitted by two female *Aedes* mosquitoes, *Aedes aegypti* (*Stegomyia aegypti*) as a main vector and *Aedes albopictus* (*Stegomyia albopictus*) (Asian tiger mosquito) with lower prevalence. The increasing number of dengue fever cases annually, affecting over 100 countries, underscores its status as an urgent health concern [30,31]. Analysis research indicates that there are approximately 390 million dengue virus infections annually, out of these cases, around 96 million lead to clinical symptoms of the disease, regardless of the level of severity [32]. According to the World Health Organization's (WHO) identification of this RNA virus, vector-borne disease with four serotypes is considered the fastest-spreading viral infection (https://www.who.int/news-room/fact-sheets/detail/vector-borne-diseases). Climate change and global warming are facilitating the growth of *Aedes* mosquitoes, contributing to the emergence of new dengue fever endemic areas not only in tropical countries but also in regions where the disease was previously not endemic [33,34]. Furthermore, dengue has not reached pandemic levels, but it is endemic in many regions and continues to cause sporadic outbreaks and epidemics, particularly in tropical and subtropical areas [31,35]. The WHO reported a significant increase in dengue fever cases, reaching 5.2 million in 2019, with many infections going unrecorded due to their asymptomatic or mild nature. Fast forward to 2023, an unprecedented surge in reported cases has sparked concerns about the virus's global spread into previously unaffected areas (Fig. 1) [36]. The dengue virus (DENV) poses a significant threat to approximately half of the global population, with its widespread geographical distribution leading to high infection rates in various regions, including Southeast Asia, the Western Pacific, the USA, and Africa, where around 70 % of cases occur in Asia [32]. In 2021, the 5 countries of Brazil, India, Vietnam, the Philippines, and Colombia have reported the highest number of cases (Table 1) [37]. Dengue fever with four confirmed serotypes (DENV-1, DENV-2, DENV-3, and DENV-4) that are transmitted to humans through a female mosquito bite is often known as "break-bone fever" and can result in symptomatic or asymptomatic viral infection [[38], [39], [40]]. Nevertheless, dengue fever is caused by four distinct serotypes; the fifth serotype (DENV-5) was isolated in October 2013 from a 37-year-old farmer who was hospitalized in Malaysia [41]. DENV-2 and DENV-1 have been identified as the most prevalent stereotypes in in America, but since 2023, there has been a notable increase in the detection of DENV-3 and DENV-4 [42]. However, most countries have recently recognized the co-circulation of all four dengue serotypes [42] (Fig. 2). The majority of dengue fever cases are mild and asymptomatic, with a short duration typically less than one week, and in cases where symptomatic forms occur, the patient may experience an acute fever lasting 3–7 days [43,44]. The phase of dengue fever illness can be classified as acute febrile phase, critical phase, and convalescent phase [45]. Initial DENV infection may present with symptoms such as fever, myalgia, headache, and joint pain, resembling those of other diseases like COVID-19, influenza, and malaria, while laboratory results typically reveal leukopenia, thrombocytopenia, and slight increases in serum hepatic transaminases [43,[46], [47], [48]]. In severe dengue illness, initial signs and symptoms include significant plasma leakage, shock, severe bleeding, elevated AST and ALT levels (≥1000 units/L), and the potential for organ failure [46]. The laboratory diagnostic techniques of dengue fever encompass a broad spectrum and due to the importance of dengue fever it is essential to obtain accurate and rapid methods. Diagnostic techniques include identifying and isolating the virus, which is crucial for identifying new serotypes and genotypes as well as understanding serotype-specific immunity [49,50]. Quantitative polymerase chain reaction (QRT-PCR), serological detection, non-structural protein 1 (NS1) antigen detection, and sequencing are commonly utilized techniques for diagnostic purposes [49]. There are currently no approved antiviral medications for treating dengue, making it essential to focus on supportive care, fluid therapy, fever management, and close monitoring of the patient [51]. The recent introduction of dengue vaccines represents a significant advancement in public health, with the first-generation vaccine, Dengvaxia® (CYD-TDV), approved in 2016 for individuals aged 9 to 45 who have previously been infected with dengue, as seronegative individuals may face an increased risk of severe illness following vaccination; however, the vaccine has limitations, including restricted usage and the risk of antibody-dependent enhancement [52]. However, the CYD-TDV vaccine demonstrates a 65 % protection rate against dengue infection [53,54]. In contrast, the second-generation vaccine, Qdenga® (TAK-003), which was approved by the European Medicines Agency in December 2022, offers broader applicability and has received approval in multiple countries; it is a live-attenuated tetravalent vaccine that stimulates immune responses to all four dengue serotypes [55]. Qdenga® is generally well-tolerated and effective in preventing dengue fever among children and adolescents in endemic regions, but it may not provide complete immunity against DENV3 and DENV4 for dengue-naive individuals, highlighting the need for further research to refine vaccination strategies for this group [56]. The most common side effects of the subcutaneous Qdenga® vaccine, affecting over 20 % of recipients, include pain and redness at the injection site, headache, muscle pain, general discomfort, and fatigue, with about 10 % experiencing fever; these mild to moderate side effects typically resolve within a few days and are generally less pronounced after the second dose compared to the first [[57], [58], [59], [60], [61], [62], [63]].

**Table 1:** Number of dengue fever cases in Brazil, India, and Colombia from 2021 until December 25, 2024.

Country	Number of cases							
	2021	2022	2023	2024	Serotype	Deaths		Ref
						2021	2024	
**Brazil**	975,474	2,363,490	3,088,723	10,107,270	DEN 1,2,3,4	239	5925	[64]
**India**	193,245	233,251	289,235	186567	DEN 1,2,3,4	346	160	[65,66]
**Colombia**	53,334	69,497	131,784	311,536	DEN 1,2,3,4	43	217	[64]

### 3.1 Climate variability and global warming

**Fig. 3:**
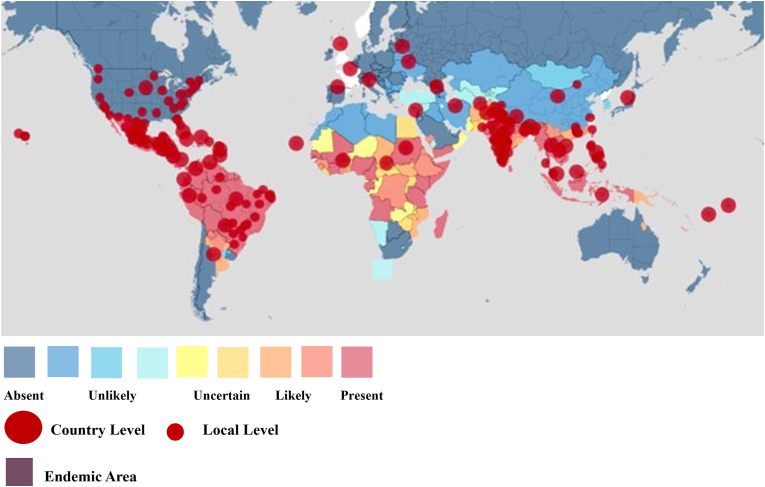
Heatmap analyses of domestic or imported dengue virus cases (June 25, 2024–December 25, 2024) (https://www.healthmap.org/dengue).

Climate variations and rising temperatures influence the pattern of dengue transmission and could potentially have a significant impact on the transmission cycle between vectors and hosts, as well as on the spread of transmission [67]. Higher temperatures can quicken the hatching of eggs and shorten the extrinsic incubation period, and humidity plays a vital role in influencing the behavioral patterns of mosquitoes, including their mating and egg-laying activities [[68], [69], [70]]. Extreme high temperatures may also have a different effect, research conducted in Singapore revealed that the risk of dengue fever decreased as the temperature increased to extreme levels (Fig. 3) [71]. In southern Thailand results specified climatic factors play an effect in transmission cycles of this illness and have demonstrated at high humidity female mosquitoes have an extended lifespan therefore, allowing them more time to feed on infected individuals [72,73]. Flavivirus structure alterations caused by elevated temperature have been indirectly detected, showing changes in the structure of the virus at an elevated temperature of 37 °C. However, these modifications do not impact the virus's ability to infect [74]. Multiple studies have identified temperature as a crucial factor influencing dengue transmission. Optimal temperature ranges for *Ae. aegypti* vector competence and disease transmission are generally between 26 °C and 29 °C. However, transmission can occur within a broader range of temperatures, from approximately 18 °C–34 °C. Higher temperatures, coupled with elevated humidity and mosquito populations, can further exacerbate transmission rates. Additionally, these findings highlight the importance of considering temperature fluctuations and climate change in predicting and mitigating dengue outbreaks [73,[75], [76], [77], [78]]. Further research indicated that higher temperatures, along with elevated humidity and a high mosquito population, led to an increase in the transmission rate of human dengue fever infection during 2001–2008 in southern Taiwan with a 3-month delay [79]. In 1987, Watts et al. investigated the effect of temperature on the capacity of *Ae. aegypti* to transmit DENV-2 to rhesus monkeys in Bangkok, Thailand, which was evaluated as a potential factor contributing to the seasonal fluctuations in the occurrence of dengue hemorrhagic fever [80]. According to the temperature variation and global warming, some studies even design dengue forecasting models using temperature and rainfall that warn during dengue epidemics [81]. Moreover, several studies have explored the impact of high temperatures on the dynamics of dengue fever; however, research findings have varied [[82], [83], [84], [85], [86], [87], [88]]. The spread of this illness could be significantly influenced by a range of climatic and socioeconomic elements, and future research suggested future changes in dengue transmission, such as the emergence of new endemic areas for mosquito vectors [89]. The temperature rise is extending the transmission season for mosquitoes, leading to a higher number of infected cases. Moreover, extreme weather conditions can impact mosquito breeding, outdoor activities of humans, and individuals living in poverty, leading to fluctuations in the number of cases reported each year [90]. The global dengue outbreak underscores the importance of effectively managing the emerging risk of dengue fever, particularly in the context of global warming [91].

### 3.2 International travel

The spread of dengue fever is significantly influenced by human transportation networks [92]. As the most significant mosquito-borne viral disease globally and the second leading cause of febrile illness among returning travelers, it has the potential to be transmitted and spread by international travelers [93,94]. The travel destination and the time since arrival from dengue fever endemic areas can aid clinicians in making more effective requests for molecular testing [95]. Moreover, the increase in international air travel between countries with endemic dengue, often popular tourist destinations, and regions without the disease has heightened the risk of transmission [96]. Although the limited flight range of primary mosquito vectors (*Ae. aegypti* and *Ae. albopictus*) initially suggested that airborne transmission between continents was unlikely, research has shown that the virus can still spread through contaminated containers carrying their eggs [[97], [98], [99]]. Interestingly, Dengue affects both adult and pediatric travelers, and among those returning from Southeast Asia, it has become a more common cause of febrile illness than malaria [100].

Modern transportation systems facilitate the rapid spread of dengue viruses and mosquitoes to various geographic areas worldwide [101]. Research on various cases in Japan from 2005 to 2010 found that Asia was the most common region of acquisition for imported dengue fever cases (90.0 %) [102]. Iran, the second-largest-country in the Middle East, reported its first case of dengue fever in 2008, involving a patient who had traveled to Malaysia [103]. Research by Atlassian et al. in Saudi Arabia revealed that human migration patterns and the influx of Muslim pilgrims significantly contribute to the transmission of dengue fever by introducing novel viral strains into the region [104]. Other study on cases of travel-acquired dengue fever imported to Denmark revealed that this disease is primarily brought into the country by adult males who have recently returned from Southeast Asian countries [105]. Baaten et al. found that travel during the rainy months in the Netherlands from 2006 to 2007 significantly increased dengue fever incidence [106]. Additionally, fatal cases of dengue in travelers from non-endemic countries were reported to involve primary infections rather than secondary ones [107]. The research on the risk of dengue virus importation into Europe through air travel in 2010 identified that the highest risk of dengue importation through air travel occurred during the months of August, September, and October, and which can be prioritized based on the number of travelers arriving from dengue-affected regions to cities where the vector *Aedes albopictus* is present [96]. A recent investigation also revealed the relationship between dengue fever and febrile cases among tourists visiting Bali [108]. A study in Italy from 2005 to 2012 identified a rising trend of dengue infections imported into Rome via air travel, corresponding seasonally with periods of increased mosquito vector activity [109]. Consequently, there is a strong emphasis on the necessity of vaccination and monitoring individuals who have traveled to infected areas or during certain seasons.

### 3.3 Rainfall and flood

Floods and precipitation have been identified as significant and influential risk factors for the reproductive processes, growth rates, behavioral tendencies, and population trends of arthropod vectors like mosquitoes, which serve as carriers for dengue [110]. Research indicates that the frequency of dengue cases in Pakistan is closely tied to rainfall levels, with infections most prevalent in flood-affected regions [111]. Similarly, flooding in Kenya has been associated with outbreaks of infectious diseases, including waterborne, rodent-borne, and vector-borne illnesses [112]. Additionally, flooding can deteriorate housing quality, particularly during hurricanes, leading to increased vulnerability and greater interaction between vectors and humans [113,114]. Statistical analysis in Indonesia (2009–2013) revealed a significant relationship between the duration of rainy days and rainfall levels with the incidence of dengue hemorrhagic fever (DHF) [115]. Research by Pinontoan et al. in Manado City indicated that although air temperatures tend to rise, there is a corresponding decline in rainfall, humidity, and instances of DHF [116]. Additionally, Santos et al. examined the relationship between rainfall and dengue cases in João Pessoa City, Brazil, finding that the number of cases increased during the initial months following the rainy season [117]. Benedum et al. introduced a non-linear methodology to analyze the connection between rainfall, flushing events, and the incidence of dengue outbreaks in Singapore [118]. Natural flood events and extreme rainfall during specific times of the year have been identified as major risk factors for the transmission of dengue fever. Therefore, it is essential to implement adequate preparations, including effective emergency medical response and timely treatment of infectious diseases, to reduce mortality rates and manage these natural disasters, thereby mitigating the rise in dengue fever cases [119].

### 3.4 World Cup, Olympic and Paralympic games

Dengue fever exemplifies the potential dangers associated with major international events, such as the World Cup and Olympic Games, which highlight the global transmission of arboviral diseases spread by arthropods. Contemporary society witnesses frequent mass gatherings, attracting immense crowds from diverse global locations. These events inherently pose a significant risk of localized amplification and subsequent global dissemination of infectious diseases. [120]. Travelers attending such gatherings may inadvertently introduce pathogens acquired from their respective origins. While host countries typically spearhead preparedness efforts for infectious disease threats at these events, often in conjunction with international public health organizations, the COVID-19 pandemic and the escalating prevalence of vector-borne diseases present novel and critical challenges, particularly for cities hosting major sporting events [60,121]. Furthermore, rising summer temperatures, exacerbated by urban heat island effects and dense spectator crowds, pose a growing risk of heat-related injuries among both athletes and spectators at international sporting competitions, as observed across various regions, from Asia to North America [120]. Risk of infectious disease outbreaks have been mentioned during past gatherings and Olympic events, as participants may unknowingly bring diseases from their local environments (Brazil, Tokyo and Vancouver) [120,[122], [123], [124]]. Additionally, exposed individuals may unintentionally carry pathogens to other regions, potentially initiating new outbreaks elsewhere [120,125,126]. Dengue fever cases have surged in the Ile-de-France Region, host of the Paris 2024 Olympics, with a notable rise in imported cases of chikungunya, Zika, and dengue. The region is home to *Ae. albopictus*, a known dengue vector, first identified in southern France in 2004 [127,128]. In 2022, France reported 65 dengue cases, which decreased to 45 in 2023, but dramatically increased to 2028 cases from May 1 to November 26, 2024 (https://www.santepubliquefrance.fr) (https://www.ecdc.europa.eu/en). Although no dengue fever cases were directly associated with the 2024 Olympic Games (26 July to 11 August), France reported a total of 68 cases of the disease between June 17 and September 8, 2024. Additionally, from January 1 to April 19, 2024, France recorded 1679 imported dengue cases—13 times more than the previous year—raising concerns as international visitors arrived for the Olympics (https://www.santepubliquefrance.fr) (https://www.fitfortravel.nhs.uk). Due to the risk of infectious disease transmission at large gatherings like the Olympic Games, proactive measures such as vaccination campaigns and comprehensive public health strategies are essential to mitigate potential outbreaks and protect the health of participants and spectators [129,130].

### 3.5 War

Environmental and social factors triggered by natural disasters, wars, and other disruptions also play a significant role in the resurgence of diseases, alongside viral and microbial influences [131]. Wars disrupt health systems and infrastructure, leading to mass population displacement and increased vulnerability to infectious diseases in overcrowded camps [132]. Historical outbreaks of typhoid, cholera, dysentery, malaria, smallpox, typhus, and influenza during and after World Wars I and II resulted in significant casualties among soldiers and civilians [[132], [133], [134]]. War creates an unstable and perilous environment for the spread of infectious diseases, increasing human exposure to the environment while limiting hygiene and access to health resources, which raises the likelihood of mosquito bites. The rise in displacement and migration, coupled with deteriorating health conditions, can be attributed to prolonged conflicts [135]. The ongoing war in Yemen has created a humanitarian crisis with millions displaced and severely compromised healthcare systems, fostering conditions for the resurgence of infectious diseases like dengue fever [136]. A study by Alghazali et al. in Taiz Governorate (July to October 2016) found that nearly half of clinically suspected dengue cases were laboratory-confirmed, highlighting the significant threat of dengue exacerbated by war-induced health service disruptions and limited public knowledge about the disease and its prevention [136]. This disease also diminishes military effectiveness, prompting the need for all nations involved to enhance research efforts and focus on developing effective vaccines and treatments [131,137].

## 4 *Wolbachia* taxonomy, epidemiology, and genomics

The significance of host specialization in the speciation processes of obligate host-associated bacteria is well established, as is the role of recombination in fostering cohesion within bacterial populations. However, it remains unclear whether divergent strains of highly recombining intracellular bacteria, like *Wolbachia*, can preserve their genetic distinctiveness while co-infecting the same host [138]. The Gram-negative bacterium *Wolbachia*, belonging to the order Rickettsiales, serves as an endosymbiont within two distinct phyla of the *Ecdysozoa superphylum*, specifically found in arthropods and nematodes from the Onchocercidae family (commonly known as Filariae) and the Tylenchida order [139,140]. From a phylogenetic perspective, the genus *Wolbachia* is divided into several distinct groups, commonly known as supergroups, each exhibiting unique characteristics and behaviors [141]. *Wolbachia* is currently classified as a single species, which is further divided into several divergent supergroups (A–N). The most extensively studied supergroups are A and B, which infect arthropods, and C and D, which infect filarial nematodes [142,143]. The supergroup classification scheme was initially proposed based on single-gene phylogenies [144], and has more recently been supported by multi-locus sequence typing [145]. Since these analyses indicated that *Wolbachia* supergroups represent genetically distinct clades, there is ongoing debate about whether some or all of these groups should be classified as separate species [8]. However, the high levels of recombination between supergroups in certain marker genes, such as the surface protein wsp [146], along with the frequent exchange of phage DNA [147], raise questions about whether the supergroup classification accurately reflects the overall genomes. Additionally, no phenotypic traits have been identified that correlate with the division of arthropod-infecting strains into different supergroups. In contrast, strains from different supergroups can exhibit similar phenotypic traits and host ranges. For instance, double infections with supergroup A and B strains occur in many insects, and both supergroups commonly induce cytoplasmic incompatibility. However, the distribution of other phenotypic traits remains less well studied [[148], [149], [150]]. Although approximately ten different *Wolbachia* strains have been identified in *Ae. aegypti* from various donor insect species, only variants of two of these strains have been successfully released and established in the field as part of replacement strategies [151]: *w*Mel [152,153] and *w*AlbB [154]. Laboratory investigations found that female mosquitoes infected with the *w*AlbB strain can become infertile as adults and may lack mature ovaries if they remain in the egg stage too long. Infertility rates can reach 75 % in females hatching from eggs stored in a humidity-controlled environment for 11 weeks [155,156].

Analysis of genomic data from 172 *Ae. aegypti* females across six populations led to a refined characterization of their microbiome, identifying 844 bacterial species across 23 phyla. Among these, 54 species were found to be ubiquitous across populations. The density of *w*Mel infection varied significantly among individuals and was negatively correlated with microbiome diversity. Network analyses showed *w*Mel as a central hub with exclusively negative interactions with other bacterial species, contrasting with a large, interconnected network of other microbiome members that likely represent the midgut community in this population [157]. Phenotypic comparisons of *w*Mel-infected and uninfected mosquitoes indicate minimal changes in the effects of *w*Mel on mosquito fitness. Antibiotic treatment to eliminate *w*Mel had limited impact on the next generation's fitness, supporting tetracycline's effectiveness for generating uninfected mosquitoes without off-target effects. *w*Mel maintains a stable within-host density and induces complete CI. Genomic comparisons from before and after release show few differences, suggesting that releases of *Wolbachia*-infected mosquitoes for population replacement will likely remain effective for years, although ongoing monitoring is essential to detect potential evolutionary changes [158]. The analysis of *Wolbachia* biology has advanced with the sequencing of genomes from many isolates, now publicly available for over 90 host species [159,160]. Additionally, mining raw genomic data from hosts has uncovered numerous partial genomes. This approach has proven particularly beneficial for studying unculturable *Wolbachia* strains [161].

Several instances have been documented in both insects and nematodes where portions of the *Wolbachia* genome, including some large segments, have been transferred to the host chromosomes [[162], [163], [164]]. These cases either represent recent events in which *Wolbachia* and host sequences are highly similar or involve significant pseudogenization [163]. Most genes transferred from *Wolbachia* to their hosts are nonfunctional in the recipient genome, as these gene copies often contain stop codons, frameshifts, or retroelement insertions, and may also be transcriptionally inactive [164,165]. Consequently, these transferred genes generally do not meet the basic criteria for evolutionary significance—longevity and integration into the biology of the recipient taxon [166]. In contrast, the family of salivary gland surface (SGS) proteins identified in *Ae. aegypti* and *Anopheles gambiae* satisfies both of these criteria. SGS genes lack known non-mosquito eukaryotic homologs but exhibit moderate sequence similarity to the hypothetical gene WD0513 from *w*Mel, the *Wolbachia* endosymbiont of *Drosophila melanogaster* [167,168]. This has led to the hypothesis that these genes originated from an ancient horizontal gene transfer, likely from *Wolbachia* to mosquitoes [168]. In the absence of strong host specialization, niche partitioning may promote speciation, necessitating genomic data to evaluate recombination and identify features explaining supergroup separation; however, preparing sufficient DNA for sequencing obligate host-associated bacteria with low infection densities remains time-consuming [169,170]. Due to these challenges, genomic data is currently available for only a limited number of *Wolbachia* strains. Analysis showed that *Wolbachia*-infected samples were mainly found within a specific mitochondrial clade, indicating a potential link between certain mitochondrial lineages and *Wolbachia* infection. The genetic diversity within the *Aedes aegypti* mitochondrial genome and its potential association with maternally inherited *Wolbachia*, considering the implications for the effectiveness of *Wolbachia*-based mosquito control strategies [171]. The slow evolutionary rate of the *16S rRNA* gene has made it difficult to create a comprehensive phylogeny for *Wolbachia* strains based solely on these sequences; however, new methodologies have emerged to address this challenge [172]. Recently, the use of the rapidly evolving cell-cycle gene *ftsZ* has shown promise in enhancing phylogenetic resolution within the *Wolbachia* clade [173]. While both *16S rRNA* and *ftsZ* gene sequences have enabled the differentiation of major *Wolbachia* groupings, they have not provided sufficient detail to clearly define the relationships among individual *Wolbachia* strains with varying reproductive traits [174]. Many *Wolbachia* genomes have been analyzed to gain insights into their symbiotic relationships [175,176]. Several potential genes have been identified as involved in CI [177,178], male-killing [179], feminization [180] and nutritional support mechanisms [181,182]. The ability of mosquitoes to carry certain traits, known as the CI phenotype, is now being utilized as a means to prevent human diseases transmitted by mosquitoes [183].

*Wolbachia*'s establishment can introduce new mitochondrial DNA variants, suggesting significant interactions between mtDNA and *Wolbachia* [184,185]. Comparing mtDNA and *Wolbachia* genomics within hosts and across species reveals instances of horizontal or introgressive *Wolbachia* transfer. These aids understanding of *Wolbachia*'s evolutionary history and spread mechanisms. Researchers can pinpoint transfer events by examining genetic similarities and differences between mtDNA and *Wolbachia* across different host species [186]. There is currently no documented evidence of widespread horizontal transmission of *Wolbachia* among mosquitoes [187]. The assembly of 110 high-quality *Wolbachia* genomes with Darwin Tree of Life project [188] demonstrates the potential of long-read data and effective analysis methods. This research revealed a tendency for different supergroups to infect various insect orders and identified several host-switching events during the *Wolbachia* pandemic. Additionally, genome size in *Wolbachia* correlates with the abundance of bacteriophage WO copies [189]. A study evaluated the efficiency and limitations of double digestion restriction site-associated DNA sequencing (ddRAD-Seq) for detecting and quantifying *Wolbachia* in field-collected *Ae. aegypti* populations in Metro Manila, comparing it to PCR-based assays. Results indicated that male *Ae. aegypti* had more reads mapped to the *Wolbachia* genome than females, suggesting higher prevalence rates in males [190]. Recent studies have examined the epidemiology of *Wolbachia* in *Ae. aegypti* [158,[191], [192], [193]]. Field trials releasing *Ae. aegypti* mosquitoes infected with the *Wolbachia w*Mel strain have shown success in several regions including Northern Australia, the USA, Malaysia, Indonesia and more recently in Southeastern Brazil [154,[194], [195], [196], [197]]. A study evaluating *Wolbachia* infection in *Ae. aegypti* mosquitoes in Medellín, Colombia found low overall prevalence (9.5–33.2 %) with highly heterogeneous distribution, varying significantly across communes, suggesting a decline in *Wolbachia*-infected mosquitoes since release efforts ended [198].

## 5 *Wolbachia* transmission, tropism and infection mechanism in dengue-carrying *Aedes* mosquitoes

*Wolbachia*, an intracellular bacterium present in over half of all insect species, has demonstrated significant success in suppressing the transmission of dengue viruses in blood-feeding arthropods, notably mosquitoes [27,194,[199], [200], [201], [202]]. Given its widespread presence among insects and mites, *Wolbachia* can disseminate rapidly within populations and across species. When it induces strong CI, local proliferation can occur quickly once its frequency exceeds a specific unstable threshold, which is expected to be near zero for naturally occurring infections but may range from 10 % to 30 % for many instances of transinfection [203]. The rapid local proliferation of *Wolbachia* indicates the significant advantage female carriers gain over non-carriers when male carriers are prevalent. Natural invasions of *Wolbachia* are rare, which is not surprising given that most attempts at horizontal transmission in the wild are likely unsuccessful; however, when successful, geographic expansion can occur swiftly [204].

Intraspecific horizontal transmission appears to be uncommon overall, aside from a few instances [186,[205], [206], [207]]. Empirical cases are showing spatial distribution associated with, male killing [208], and scenarios without reproductive manipulation [205,206]. *Wolbachia* variants inducing strong CI typically dominate populations at high frequencies [209]. Conversely, *Wolbachia* strains that exhibit male-killing traits generally maintain lower prevalence levels within populations, although exceptions do exist [210]. The propagation of *Wolbachia* can be inhibited by different strains of the same bacteria. Specifically, in the mosquito species *Culex pipiens* found in Tunisia, there exist two distinct variants of *Wolbachia* that exhibit a noticeable geographical segregation. These variants maintain a distinct boundary that has remained unchanged for seven years. Interestingly, one variant induces a strong CI effect when crossed with females harboring the other variant. Conversely, crosses involving the reciprocal combination result in CI only in certain instances [211].

The *Ae. aegypti* WB1 line was previously established through the microinjection transfer of the *Wolbachia* strain *w*AlbB from *Ae. albopictus* into the wild-type *Ae. aegypti* Waco strain [202]. *Wolbachia* strains like *w*MelPop, *w*Mel, and *w*AlbB have proven effective in trans-infecting *Aedes* mosquitoes. However, the *w*Mel-*Wolbachia* strain is predominantly utilized due to its widespread success [13,154,212,213]. The deployment of *w*Mel *Wolbachia* has shown significant success in mitigating the population of mosquitoes that carry dengue fever; however, this approach comes with inherent risks [214]. One such risk stems from challenges in establishing or maintaining stable populations of *Wolbachia*-infected mosquitoes, particularly because these mosquitoes struggle to pass *Wolbachia* vertically to their offspring in environments with high temperatures [215,216].

The WB1 strain of *Ae. aegypti* demonstrates significant resistance to the dengue virus in comparison to its parent strain, Waco [217]. The *w*AlbB strain prevented viral replication within the mosquito's midgut and its spread through the thorax and head, significantly lowering the mosquito's capacity for virus transmission. Observations of resistance against various arboviruses were noted in *Ae. aegypti* mosquitoes carrying the *Wolbachia* strain *w*MelPop-CLA [28], as well as in *Culex quinquefasciatus* mosquitoes infected with *w*Pip [218]. The recent findings regarding *Wolbachia*-induced impacts on mosquito vectors present significant possibilities for implementing population replacement tactics [219].

*Wolbachia* infection diminishes the overall health and reproductive capabilities of *Aedes* mosquitoes, impacting their survival rates. This effect is primarily due to CI, leading to sterility in male mosquitoes carrying the infection and reduced egg production in infected females compared to uninfected ones. Consequently, this diminished fitness challenges the mosquito population's self-sustainability, effectively managing the population size. *Wolbachia*'s spread within mosquito populations hinges on this critical mechanism. Conversely, when an infected female mates with an uninfected male, the offspring are capable of surviving but inherit the *Wolbachia* infection. This strategy facilitates the widespread dissemination of *Wolbachia* among mosquito populations [220].

**Fig. 4:**
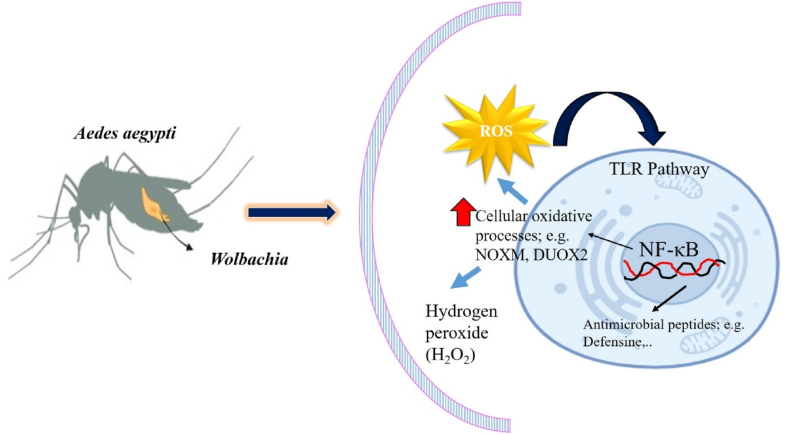
*Wolbachia* infection mechanism in dengue-carrying *Aedes* mosquitoes.

*Wolbachia* has demonstrated an ability to disrupt the replication of the dengue virus within its mosquito host. This disruption is believed to happen through several methods, such as competing for resources within the mosquito cells and triggering immune reactions in the mosquito that prevent viral replication. Consequently, mosquitoes infected with *Wolbachia* are less effective at transmitting the dengue virus to humans. The molecular basis behind *Wolbachia*-mediated resistance to pathogens is not entirely clear, but it seems to involve enhancing the mosquito's innate immune system [28,200,201,217]. The infection caused by this bacterium triggers oxidative stress and raises the level of reactive oxygen species (ROS) within its mosquito host. An increase in ROS levels correlates with the initiation of the Toll pathway, a crucial mechanism for inducing antioxidant production to mitigate oxidative stress. Additionally, this immune response pathway plays a role in activating antimicrobial peptides such as defensins and cecropins. [28,217,221]. In line with these findings, it has been demonstrated that activating the Toll pathway through RNA interference (RNAi) to deplete cactus, a suppressor of REL1, inhibits the proliferation of dengue in the mosquito species *Ae. aegypti* [222]. ROS plays a pivotal role in activating the Nuclear Factor-Kappa B (NF-ĸB) pathway, which is fundamental to the body's immune response, inflammation processes, and the preservation of cell survival. This activation is influenced by the balance between ROS production and antioxidant defenses within the cell, affecting both the initiation and resolution phases of inflammation [223,224]. In some cell types, exposure to hydrogen peroxide (H_2_O_2_) or other oxidants leads to the movement of NF-κB into the nucleus [225,226]. Conversely, the interaction between ROS and the NF-κB pathway also involves the activation of antioxidant gene expression, which helps protect against ROS damage [223]. This protective mechanism is characterized by an increase in the expression of several antioxidant genes, including manganese superoxide dismutase (MnSOD), copper-zinc superoxide dismutase (CuZnSOD), ferritin heavy and light chains, glutathione peroxidase (GPX), and catalase, in WB1 mosquitoes. The infection of *Ae. aegypti* with *Wolbachia* results in a significant increase in the expression of genes that encode for the NOXM and DUOX2 enzymes, which are crucial for generating ROS [[227], [228], [229]]. Additionally, a higher concentration of H_2_O_2_ was observed in WB1 compared to Waco mosquitoes [229]. This elevation mirrors the pattern found in parasitic *Rickettsia* bacteria, which also exhibit high levels of H_2_O_2_ as a primary oxidant, leading to cellular harm. Elevated ROS levels can harm cellular structures, inducing oxidative stress within the host [230]. The detrimental impact of ROS on organisms is mitigated by an antioxidant defense mechanism (Fig. 4).

## 6 *Wolbachia* intracellular proliferation and survival in the host

**Fig. 5:**
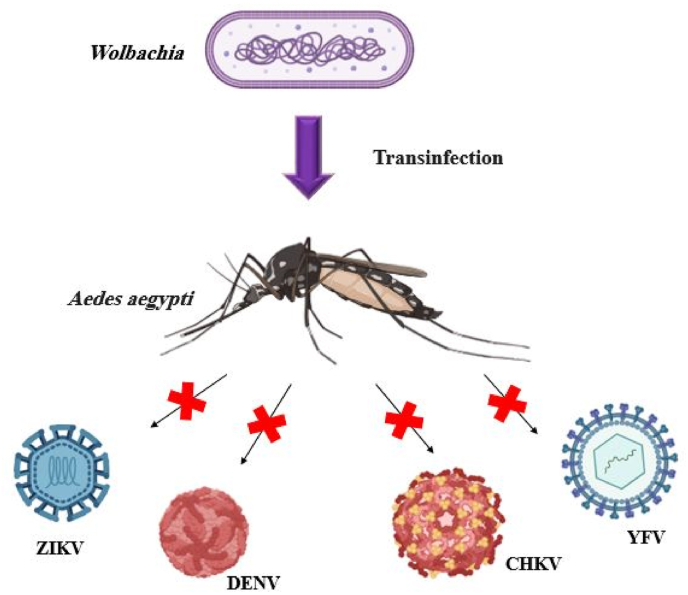
Transinfection of *Aedes aegypti* with *Wolbachia. Aedes aegypti* infected with *Wolbachia* loses the ability to transmit viruses to humans. Zika virus (ZIKV), Dengue virus (DENV), Chikungunya virus (CHKV), and yellow fever virus (YFV) [236].

*Wolbachia* possess a unique characteristic - their ability to inhabit and influence cellular and reproductive processes in invertebrates. This capacity likely stems from their extensive evolutionary history as intracellular organisms. What sets *Wolbachia* apart is the range of manipulations they employ. Interestingly, only another bacterial group, the genus *Candidatus Cardinium* belonging to the Bacteroidetes class, has been observed to exhibit a comparable diversity in reproductive manipulations. This distinction highlights the remarkable adaptability and specificity of *Wolbachia*'s interactions with host organisms [231]**.** The mechanisms behind *Wolbachia*-mediated pathogen blocking remain unclear, though two primary theories have emerged. These include immune priming and competition between symbiotic bacteria and pathogens for host resources. Recent studies indicate that *Wolbachia* enhances immune gene expression in infected vectors, particularly where pathogen blocking is most pronounced. Interestingly, research suggests that immune activation may not be crucial, as certain native *Wolbachia* strains fail to induce these immune responses yet still provide some level of protection against pathogens. This finding challenges our understanding of how *Wolbachia* exerts its protective effects and highlights the complexity of this phenomenon [232]. *Wolbachia* restricts the replication of various viruses (dengue, yellow fever, Zika, West Nile, and Chikungunya) additionally, there are reports concerning the impact of bacteria on filarial nematodes and the malaria-causing parasite *Plasmodium* within the specific mosquito carriers (Fig. 5). Numerous investigations have been conducted regarding the function of *Wolbachia*-induced changes in immunity gene expression, the increases in immune gene expression take place as a response of the host to infection and context and exert influence over the antioxidant systems of its host, facilitating *Wolbachia* own survival [[233], [234], [235]].

The enduring symbiotic relationship between *Wolbachia* and an *Ae. albopictus* cell line is characterized by the production of ROS and the activation of antioxidant enzymes [142]. The enhancement of ROS has been observed alongside the limitation of DENV (dengue virus) infection via the Toll pathway. This correlation suggests that *Wolbachia* may exert anti-DENV effects by stimulating the Toll pathway. This mechanism implies a potential role for *Wolbachia* in modulating the host's immune response against dengue virus infection, possibly through the activation of the Toll signaling cascade [229]. Moreover, *Wolbachia* can modulate the host's defensive systems, facilitating its persistent colonization and compromising the mosquito's capacity to serve as a reservoir for human pathogens [229]. *Wolbachia* also can withstand both extracellular and intracellular immunity of the host. Upon proliferation within a new host, the innate immune system is triggered through specific pathogen-associated molecular patterns (PAMPs). This activation leads to the production of antimicrobial substances, such as defensins, which result in either a reduction in *Wolbachia* levels or complete eradication of the bacteria. These effects were observed in *Ae. aegypti* mosquitoes infected with various strains of *Wolbachia*, including *w*MelPop*-CLA*, *w*Mel, and *w*AlbB [217,221,232,237]. Stable transinfection of mosquitoes has been observed to induce immune stimulation, which has been proposed as a potential mechanism for antiviral effects [28,217,229]. Research has revealed a significant connection between ROS and Toll induction in mosquitoes. This link was demonstrated through an experiment involving *Wolbachia*-uninfected *Ae. aegypti* mosquitoes. When these mosquitoes were provided with sugar water supplemented with hydrogen peroxide (H_2_O_2_), genes associated with the Toll pathway showed increased activity. Conversely, studies found that knocking down enzymes responsible for ROS production using RNA interference led to decreased expression of Toll-related genes. Under this model, researchers propose that DENV inhibition is mediated by antimicrobial peptides (AMPs) induced by the Toll pathway, such as cecropins and defensins [238].

*Wolbachia* possess a number of interesting adaptations to navigate the eukaryotic cell. Various cellular structures and organelles (the centrosome, the endoplasmic reticulum, microtubules, and the Golgi apparatus) demonstrate a significant correlation with *Wolbachia* [239]. Research has revealed that *Wolbachia* bacteria are enveloped by two membranes of bacterial origin, which are separated by a periplasmic space. These structures are further associated with a third membrane derived from the host organism [9]. Interestingly, studies have demonstrated a close relationship between *Wolbachia* and the endoplasmic reticulum, suggesting that the bacteria obtain essential amino acids from this organelle [240]. This association highlights the intricate interaction between *Wolbachia* and cellular components, particularly the endoplasmic reticulum, which plays a crucial role in protein synthesis and metabolism [11]. These interactions between bacteria and cellular organelles play crucial roles in both bacterial replication and survival. Specifically, certain organelles serve as sources of membrane material for bacterial reproduction, highlighting the intricate relationship between bacteria and host cells [241]. Additionally, the cytoskeleton and centrosomes within the host cell contribute to defining the path of bacterial movement, suggesting a complex interplay between bacterial locomotion and cellular structure [242]. This multifaceted interaction underscores the sophisticated nature of bacterial-host cell relationships and the various mechanisms employed by bacteria to ensure their survival and propagation within the host environment. Despite the restricted repertoire of amino acids and nucleotides synthesized by *Wolbachia*, this bacterium has evolved various strategies to support its metabolic needs. Specifically, *Wolbachia* possesses an array of transport proteins (importers) and proteolytic enzymes, alongside pathways for heme synthesis, riboflavin metabolism, and FAD production. These capabilities enable *Wolbachia* to generate essential nutrients for its host organism [243]. The presence or absence of particular gene products has been identified as crucial factors in symbiotic interactions [244]. Specifically, the presence of *Wolbachia* in insects can significantly impact their ability to harbor and potentially influence microbial pathogens. This influence extends to affecting the insects' capacity to transmit medically significant pathogens to humans [245]. Recent research has uncovered significant insights into the relationship between *Wolbachia*, iron metabolism, and cellular processes. It appears that *Wolbachia* may play a crucial role in regulating ferritin expression and iron metabolism within insect cells. Furthermore, studies suggest that iron homeostasis could be intricately linked to the regulation of oxidative stress. This connection is particularly noteworthy because oxidative stress is known to be a key factor influencing programmed cell death, or apoptosis [246].

## 7 *Wolbachia* interaction with the mosquitos' behaviors

### 7.1 Typical behavior of mosquito

Understanding the typical behaviors of *Aedes* mosquitoes can serve as a valuable framework for assessing the effects of *Wolbachia* infection on these behaviors and assessing the potential effectiveness of *Wolbachia* in vector control strategies. The feeding preferences of *Aedes* species can vary considerably. *Ae. aegypti* is generally considered to be primarily anthropophilic, while *Ae. albopictus* tends to exhibit more zoophilic feeding habits [247]. However, these feeding patterns are not strictly species-specific; there is evidence that *Ae. aegypti* may show a preference for avian or other animal hosts, while *Ae. albopictus* has also been noted to prefer human hosts predominantly [[248], [249], [250]]. Host preferences can be influenced by factors such as seasonal variations and human population density [251]. Both *Ae. aegypti* and *Ae. albopictus* have exhibited a bimodal feeding pattern in Africa [252,253]. *Ae. aegypti* is primarily known to bite around sunset and at night, with a smaller peak occurring in the morning [247,[254], [255], [256], [257]]. Similarly, *Ae. albopictus* also has a peak biting time in the late afternoon [252]. Although there are varying reports regarding the preferred biting locations of *Ae. aegypti*—both indoors and outdoors—*Ae. albopictus* is predominantly exophilic, with a marked tendency to bite outdoors [247]. The population of *Aedes* mosquito correlates positively with wet seasons. They are more prevalent in urban areas [247]. They prefer to breed in close proximity to human habitations including water storage containers and discarded tires [258]. Females seek areas with standing water, preferably clean and with minimal decomposing organic matter, for oviposition. *Aedes* typically seek humid, sheltered environments near breeding sites to minimize exposure to light and wind [259].

Evidence indicates that male body size does not affect mating success with large females. However, male body size significantly impacts mating success when males are paired with small females. In controlled laboratory experiments, small *Ae. aegypti* females tend to prefer small males for mating [260]. Male seminal fluids induce physiological and behavioral alterations in female mosquitoes [261]. Females typically develop resistance to further mating within 2 h after their latter copulation. However, they may still mate during this time, potentially transferring seminal fluids, although this rarely leads to the storage of additional sperm. Sperm can be stored in the female mosquito for over 3 months. The success of insemination can be affected by factors such as previous mating history, age, and body size of both male and female mosquitoes. A female mosquito can experience multiple inseminations before becoming resistant to further mating [262].

### 7.2 Impact of *Wolbachia* on mosquito behavior

#### 7.2.1 Feeding, host-preference, and biting pattern

Studies reported no significant difference in the blood meal size taken by *Ae. aegypti after infection with Wolbachia.* Interestingly, while *Wolbachia* infection does not affect the blood meal intake in *Ae. aegypti*, it is associated with a decrease in blood meal excretions in them [263]. Moreira et al. research revealed that, *Ae. aegypti* mosquitoes exhibit a prolonged pre-probing period following infection, with this duration increasing as the mosquitoes age. Older infected mosquitoes also demonstrate a more pronounced delay in probing. Additionally, infected mosquitoes, particularly at older ages, have a lower success rate in obtaining blood meals compared to their uninfected counterparts. Infected mosquitoes also show a higher incidence of abnormal feeding behaviors, such as a bent proboscis and unsteady movements, which contribute to feeding failures [264]. *Wolbachia* infection leads to a reduction of the host-seeking activity of *Ae. Albopictus. No strong evidence was found indicating that infection alters biting patterns* [265].

#### 7.2.2 Population

*Wolbachia* infection results in increased pupal production (indicating improved larval production) and shorter larval stage duration in *Ae. albopictus* population [266]. It is noteworthy that the increased pupae production may result from elevated mating rates rather than being solely attributable to *Wolbachia* infection. What's more, *Wolbachia*-infected *Ae. aegypti* larvae have been reported to manifest reduced competitiveness and slower behavioral responses to light compared to the uninfected [267].

#### 7.2.3 Mating & oviposition

Considerable increase in mating rates and efficiency in competing for wild female mosquitos in male *Ae. albopictus* infected with *Wolbachia* has been reported [265,266]. This is true even though the infection does not seem to hinder male *Ae. aegypti* from successfully mating with multiple females [268]. While the *Wolbachia* infection generally reduces fecundity and fertility in female mosquitoes, the incidence of *Ae. aegypti* females' re-mating significantly increases when mating with infected males [261,268]. *Wolbachia* infection tends to delay oviposition in *Aedes aegypti.* In contrast, it does not appear to alter the ovipositional behaviors of *Aedes albopictus* [263,265].

As previously discussed, body size influences mating success in *Ae. aegypti*, particularly among small females. A consistent pattern in laboratory settings shows that small infected males have a 20 % mating advantage over large uninfected males. However, small uninfected males have an even greater advantage, with a mating success rate approximately 27 % higher than that of large uninfected males [260].

## 8 *Wolbachia* in other hosts

*Wolbachia* are known to infect a variety of arthropods, such as mosquitoes [269]. Although researchers are exploring the possibility of utilizing *Wolbachia* to manage nematode populations, the natural prevalence and potential for manipulation of these bacteria within these organisms remain less comprehensively understood [270]. *Wolbachia* is known to infect a diverse range of arthropods, including fruit flies, butterflies, and beetles; however, the precise impacts of *Wolbachia* on these species and their possible applications in vector control remain inadequately explored [[271], [272], [273], [274], [275], [276], [277], [278]]. The symbiotic relationships between *Wolbachia* and its hosts vary depending on whether the host is an arthropod or a nematode; while *Wolbachia* can exhibit parasitic behavior, it also serves as an obligate mutualist in certain contexts, such as in the bedbug *Cimex lectularius*, where it enhances the insect's nutrition by providing essential B vitamins that are otherwise lacking in its diet, thereby establishing a form of nutritional mutualism [141,181,279]. Compared to *Wolbachia* genomes found in arthropods, those from filarial nematodes are generally smaller in size [280]. Specifically, the genome sizes range from approximately 957,990 base pairs (bp) for *w*Oo to around 1,080,084 bp for *w*Bm. This contrasts with the sizes observed in arthropod hosts, where the genomes can be significantly larger, 1,250,060 bp for *w*Mel, isolated from *Drosophila melanogaster*, 1,267,782 bp for *w*Cle, from *Cimex lectularius*, 1,587,994 bp for *w*Pip, from *Culex pipiens*, and 1,801,626 bp for *w*Fol, from *Folsomia candida* [148,281]. Furthermore, these filarial nematode-associated *Wolbachia* genomes are characterized by the presence of fewer transposable elements, including insertion sequence (IS) elements and group II intron-associated genes. Additionally, they contain fewer prophage-related genes and repeat-motif proteins, such as ankyrin domains, compared to their counterparts in arthropods [148,281]. The beta-binomial analysis of data from 172 species of fig wasps revealed a *Wolbachia* prevalence distribution with significant peaks at both ends of the spectrum, notably exceeding an 80 % incidence rate, which is substantially higher than previous estimates of *Wolbachia* infection rates across arthropods [282]. The discrepancies observed between the evolutionary histories of *Wolbachia* and its host organisms, or between *Wolbachia* and the mitochondrial DNA (mtDNA) haplotypes of its hosts, indicate instances of horizontal transmission. This phenomenon is evident across various insect orders and even within individual species [283]. Intraspecific transmission instances encompass herbivory-mediated transfer in whiteflies and horizontal transmission within *Trichogramma* species [205,206]. In species that have been extensively studied, particularly within the genera *Drosophila* and *Culex*, there is a nearly perfect alignment between the evolutionary histories of their mitochondrial DNA and *Wolbachia* infections. This high degree of congruence indicates that instances of horizontal transmission—where *Wolbachia* is transferred between unrelated hosts—are relatively uncommon, at least among these organisms [187]. It is anticipated that certain strains of *Wolbachia*, which infect arthropods, have developed symbiotic relationships with their hosts. This expectation aligns with their vertical transmission, which often favors mutualism. Research has revealed intriguing examples of *Wolbachia*'s beneficial effects on its hosts. For instance, studies have shown that *Wolbachia* infection confers protection against RNA viruses in the fruit fly *Drosophila melanogaster*. Additionally, *Wolbachia* exhibits a wide range of influences on their hosts, suggesting complex manipulations of the insect's behavior and physiology. These effects include *Wolbachia*-associated alterations in mating preferences and altered responses to olfactory cues, demonstrating the intricate nature of these microbial-host interactions [284,285]. Recent cytological studies on *Wolbachia* in *Drosophila* have uncovered intriguing interactions between this bacterial parasite and the fruit fly's cytoskeleton. *Wolbachia* has been found to exploit the host's molecular motors to facilitate its movement within the host cell. During embryogenesis, these bacteria maintain a close association with centrosomes and microtubules organized by the centrosomes. As each nuclear division occurs, approximately half of the *Wolbachia* population migrates to each spindle pole, ensuring an equal distribution of the bacteria across both daughter cells during mitosis. This remarkable mechanism allows *Wolbachia* to achieve consistent segregation throughout the development process, highlighting the complex relationship between the bacterium and its host's cellular machinery [162]. *Wolbachia* may play a crucial role as a nutritional mutualist for female *Drosophila*, particularly in low and high-iron nutritional environments. Research has shown that *Wolbachia* infection can positively affect the fecundity of female fruit flies when they are reared in conditions with limited or excessive iron intake. This discovery reveals a previously unrecognized aspect of the *Wolbachia*-*Drosophila* relationship, where the bacterial symbiont provides a fitness benefit to its host beyond its known reproductive manipulations. By enhancing fecundity in challenging nutritional scenarios, *Wolbachia* may contribute to the survival and success of infected *Drosophila* populations, potentially explaining how certain *Wolbachia* strains can successfully invade insect populations despite lacking the ability to modify host reproductive systems [286]. Correa et al. indicated that the age of the host, immune response to infection, temperature, and nutrition have significant effect on *Wolbachia* densities in female and male *Drosophila* and may be regarded as limiting factors [287]. Unlike broader immune activation, *Wolbachia*-mediated antiviral defense in *Drosophila* does not induce widespread immune stimulation, whether in naturally infected or heterologously transinfected flies [235,288]. Recent studies indicate that *Wolbachia* may acquire amino acids through the proteasomal activity of its host, a process previously observed in *Legionella pneumophila* using a mutant deficient in the AnkB F-box effector protein. The *w*Bm strain of *Wolbachia pipientis* establishes an obligatory symbiotic relationship with the filarial nematode *Brugia malayi*, which is the causative agent of lymphatic filariasis. As an obligatory endosymbiont, *w*Bm is likely to supply vital metabolites to its host. Although *w*Bm possesses a greater number of metabolic genes compared to *Rickettsia* spp., its biosynthetic functions seem to be restricted. In contrast to *Buchnera* spp., *w*Bm is capable of synthesizing only one amino acid, meso-diaminopimelate (meso-DAP), which is crucial for peptidoglycan formation. In the majority of bacteria, meso-DAP serves as an intermediate in the biosynthesis of lysine. However, similar to *Rickettsia* spp., *w*Bm does not produce meso-DAP decarboxylase (LysA), which inhibits the conversion of meso-DAP into lysine. This constraint implies that *w*Bm depends on its host for additional amino acids, which may be sourced from the host's proteasomal activity [289,290].

## 9 Progress of using *Wolbachia* for *Aedes* species

**Fig. 6:**
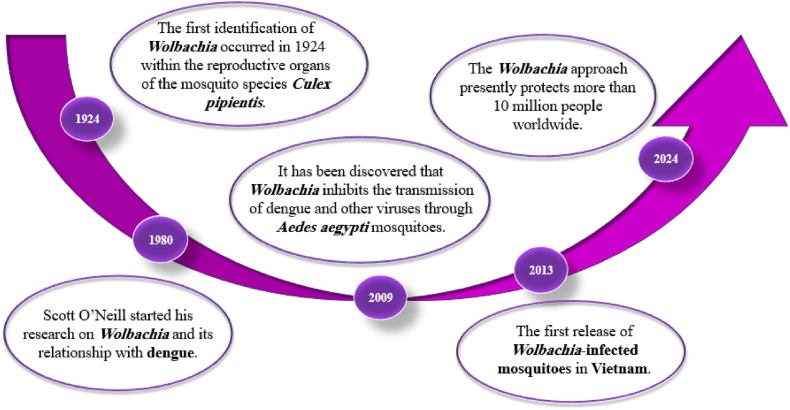
*Wolbachia* and dengue fever timeline (https://www.worldmosquitoprogram.org)**.**

The employment of bacteria in controlling dengue virus prevalence has led to the utilization of these microorganisms in specific endemic areas (Fig. 6). Strategies utilizing *Wolbachia* for managing arthropod-borne diseases primarily focus on replacing existing *Wolbachia* populations within target species with a specific strain that confers disease resistance. This approach, by altering the reproductive biology and immune response of the vector, can effectively reduce the transmission of diseases like dengue fever. While reducing the overall abundance of bacterial infections is not the primary goal, it can be a secondary effect in certain contexts, particularly when the introduced *Wolbachia* strain has a competitive advantage over native strains [291], [292]. A field study conducted by Nazni et al. in six distinct locations within the Greater Kuala Lumpur region of Malaysia investigated the establishment of the *w*AlbB strain of *Wolbachia* in *Aedes aegypti* mosquito populations. The researchers observed varying degrees of success across these sites. In some locations, the *w*AlbB strain successfully established itself and maintained high population frequencies. In other locations, while experiencing fluctuations in abundance, the strain persisted following multiple releases. This research provides valuable insights into the ecological dynamics and spatial heterogeneity of *Wolbachia*-infected mosquito populations within urban environments [154]. Ryan et al. in Australia conducted an Empirical analysis that indicated that *Wolbachia* can be effectively integrated into local mosquito populations. This integration can be achieved through various deployment strategies and within relatively brief release periods, with an average duration of 11 weeks and a range spanning from 2 to 22 weeks [293]. In Yogyakarta City, Indonesia Indriani et al. research indicates a marked decrease in dengue cases after the successful integration of *Wolbachia* into indigenous *Ae. aegypti* populations within an endemic region [294]. In two areas of Rio de Janeiro, Brazil, where *Wolbachia* will settle in the future, Dutra et al. released *Wolbachia*-uninfected Brazilian mosquitoes to investigate the impact of various urban environments on the survival rates of mosquitoes. The study indicated that the *w*Mel strain of *Wolbachia* exhibits the necessary traits for successful dissemination across various urban socio-demographic contexts in Rio de Janeiro [295]. Also, in another study in Yogyakarta, Indonesia, Tantowijoyo et al. reported the effective establishment of *w*Mel strain of *Wolbachia* and can be effectively introgressed following temporary releases of *w*Mel-infected eggs or adult mosquitoes. Furthermore, study indicated this method is well-received by local communities and that *Wolbachia* persists within the mosquito population after its introduction [196]. In Hon Mieu Island in central Vietnam, Jeffery et al. reported it would be essential to implement a preintervention control program to prevent any net rise in mosquito populations [296]. Cheong et al. in Mentari Court, Malaysia observed that mosquitoes infected with *Wolbachia* exhibited a significant infection rate four years' post-release and there were slight influences of floors and blocks on the prevalence of *Wolbachia* and eventually there was no indication that the population of *Ae. albopictus* expanded throughout the region [297]. Numerous studies have been carried out in countries including Mexico, Taiwan, Thailand, and the United States, focusing on different aspects of utilizing *Wolbachia* to manage vector insect populations and combat the dengue virus [[298], [299], [300], [301], [302]]. Several uncertainties and questions persist regarding the application of bacteria in endemic regions, necessitating comprehensive investigation and research.

## 10 Challenges of *Wolbachia* using and future perspective

*Wolbachia* field trials as a microbial pesticide have been conducted in some countries (Australia, US, Thailand and China) [29,219,303,304]. Research has examined the prevalence and variety of symbionts across all significant insect classes, including mosquito genera [305,306]. Extensive use of this method requires comprehensive knowledge of the population densities and dispersion patterns of mosquitoes at both local and regional levels. It mentioned that the density of *Wolbachia* in transinfected mosquitoes, plays a vital role in the antiviral activity mediated by *Wolbachia*, and reduction in *Wolbachia* density resulting from the mosquito/*Wolbachia* co-adaptation could result in a notable decline in antiviral efficacy [157,270]. Although *Wolbachia*-based systems have demonstrated their effectiveness, further extensive research is required in these areas. One important aspects of future research involve unraveling the intricate and evolving characteristics of *Wolbachia* across various insect orders. An additional significant aspect of the investigation into this bacterial weapon is *Wolbachia* host-shifting. Conversely, concerns regarding its effects on human diseases must be considered [307,308]. Due to the faster mutation of the virus compared to mosquitoes and bacteria, new variants are created that are capable of replicating in *Wolbachia*-carrying mosquitoes may be rapidly selected, and may persist in *Ae. aegypti* population which is infected by *Wolbachia* as long as they are capable of effectively replicating within the human host [309]. To manage mosquito populations, adult males infected with artificially induced *Wolbachia* have been released to breed with wild female mosquitoes leading to a reduction in the population of the target species over time. Conversely, the fitness impacts of *Wolbachia* on new hosts are challenging to anticipate due to significant variations in *Wolbachia* densities and tissue distributions when comparing native and novel hosts [310]. Although the antiviral properties of *Wolbachia* have been indicated in studies, there are many questions and assumptions. The antiviral properties observed are probably associated with an elevated density of *Wolbachia* and may also involve immune activation in the novel host, however, direct evidence is currently insufficient [24]. *Wolbachia*-resistance virus is another challenge discussed in this area. The rapid mutation rate of DENV virus adapts to the selective pressures imposed by *Wolbachia*, ultimately leading to resistance against this intervention [309]. The dengue virus exhibits a capacity for genomic mutation, leading to the emergence of novel strains. These successive alterations may result in the emergence of strains that the *Wolbachia* is incapable of defending against and various strains exhibit distinct and unique behaviors within the vector, which may lead to the unpredictability of the behavior of the strains [29]. Assessing the epidemiological impact of deploying *Wolbachia* to mitigate the transmission of dengue virus presents significant challenges [311]. Alternative bacterial systems exist that can produce phenotypes typically linked to *Wolbachia*, these systems may be more prevalent than *Wolbachia* itself [312]. Despite the comprehensive explanations provided, numerous inquiries remain regarding the large-scale application of this bacterium. PCR-based techniques are frequently employed to identify *Wolbachia* in *Ae. aegypti*; however, they may not be the most suitable choice for extensive field research. Although these assays are quick and straightforward, they are susceptible to false negative outcomes, especially when dealing with arthropod samples [313]. Conventional PCR, designed to amplify a specific target gene, cannot differentiate between a bacterial gene that has been integrated into the mosquito genome and a genuine, active *Wolbachia* infection [314]. While allele-specific PCR is recognized as a reliable technique for amplifying *Wolbachia* DNA, the occurrence of false positive and false negative results has not been thoroughly investigated [315,316]. These challenges and constraints necessitate further in-depth and extensive research into the various aspects of utilizing *Wolbachia*.

## 11 Conclusion

Diseases that can be transmitted through insects are one of the most important concerns of humanity, and research aimed at their prevention and treatment continues to be a priority. Dengue fever has emerged as a serious public health concern in recent years. The alterations in the prevalence pattern of this disease, coupled with the rising number of reported cases, require more extensive investigation focused on strategies for prevention and treatment. *Wolbachia*, an obligate endosymbiotic bacterium, is capable of infecting many insect species in a wide range and is considered environmentally safe. *Wolbachia*-based control of dengue fever is non-pathogenic in humans and research conducted across various generations of mosquitoes indicates that the risk of increasing resistance to *Wolbachia* within the mosquito's body is minimal. Further studies are required to investigate the exact molecular mechanism of transmission from one host to another, precise forecasting models for the large-scale utilization of *Wolbachia*, *Wolbachia*-infected mosquitoes release into the wild population of mosquitoes, virus develop the ability to overcome the protective effects of *Wolbachia* bacteria and the long-term viability and effectiveness of using *Wolbachia* bacteria to control mosquito populations. The field of *Wolbachia* symbiosis is undergoing rapid development and promising opportunities for its application are on the horizon.

## CRediT authorship contribution statement

**Sahel Safaei:** Writing – original draft, Conceptualization. **Mozhgan Derakhshan-sefidi:** Writing – original draft, Investigation, Conceptualization. **Amirmohammad Karimi:** Writing – original draft.

## Consent for publication

Not applicable.

## Data availability

The datasets used and/or analyzed during the current work are available upon reasonable request from the corresponding author.

## Funding

No special finding received for this work.

## Declaration of competing interest

None.
